
JOURNAL INFORMATION
==============================
NLM Title Abbreviation: ScientificWorldJournal
Journal ID: ScientificWorldJournal
NLM Journal ID: 101131163
Journal ID: TSWJ

The Scientific World Journal

ISSN: 2356-6140
EISSN: 1537-744X
Publisher: Wiley

ARTICLE INFORMATION
==============================
PMCID: PMC6009627
PMID: 29973810
DOI: 10.1100/tsw.2002.36
Article ID: 735345
Article version: 1
Subjects: Short Report

HRAD30A, a Gene Encoding a Class Y Low-Fidelity Dna Polymerase Has a Low Complexity Region in Intron I Associated with Tissue-Specific Alternative Splicing

Cleaver James E. *
Thakur Manish
Volik Stas
Collins Colin
UCSF Cancer Center, University of California, San Francisco, CA 94143-0808, USA
*James E. Cleaver: jcleaver@cc.ucsf.edu
Publication date: 2002
Electronic publication date: 2002 Jan 18
Volume: 2
First page: 75
Last page: 76
Copyright: Copyright © 2002 James E. Cleaver et al.
Copyright year: 2002
License: This is an open access article distributed under the Creative Commons Attribution License, which permits unrestricted use, distribution, and reproduction in any medium, provided the original work is properly cited.
License URL: https://creativecommons.org/licenses/by/3.0/

Funding: National Institute of Environmental Health Sciences ES/CA08061 Ellison Foundation

==============================
Advances in Gene Technology: The Genome and Beyond –
Structural Biology for Medicine (Proceedings of the 2002 Miami
*Nature Biotechnology* Winter Symposium)
*TheScientificWorld* 2002, 2(S2), 75–76
ISSN 1532-2246; DOI 10.1100/tsw.2002.36

### *HRAD30A*, A GENE ENCODING A CLASS Y LOW-FIDELITY DNA POLYMERASE HAS A LOW COMPLEXITY REGION IN INTRON I ASSOCIATED WITH TISSUE-SPECIFIC ALTERNATIVE SPLICING

James E. Cleaver, Manish Thakur, Stas Volik, and Colin Collins
UCSF Cancer Center, University of California, San Francisco, CA 94143-0808
jcleaver@cc.ucsf.edu

**INTRODUCTION**. The XP Variant (XPV) is a complementation group of the human disease xeroderma pigmentosum that was recently identified as a caused by mutations in a gene that maps to 6p21, *hRad30A*, that encodes a novel DNA polymerase, pol η[1,2]. Pol η is a member of a large family, class Y, of low-fidelity DNA polymerases. It has the unusual property that although it can replicate UV-induced pyrimidine dimers *in vivo* with the insertion of the correct bases in the daughter strand, *in vitro* it is very error-prone and inserts mutagenic bases at about 1% frequency[3,4]. We recently identified an alternative splice variant of *hRad30A* mRNA in the testis and fetal liver, suggesting that there may be tissue-specific regulation of this gene[5]. This alternative splicing results in the elimination of exon II that contains the ATG start site for transcription and results in a cDNA that does not complement the XPV defect[5].

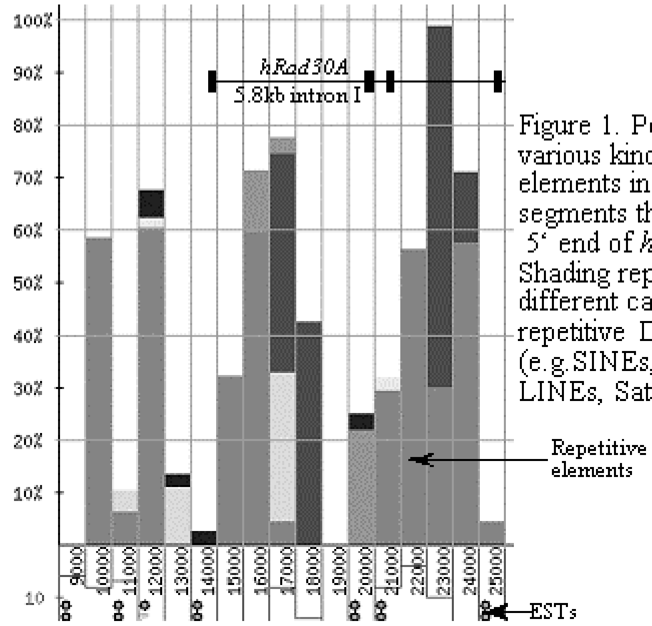

Figure 1. Percentage of various kinds of repetitive elements in 1kb segments through the 5' end of *hRad30A* Shading represents different categories of repetitive DNA (e.g. SINEs, LTRs, LINEs, Satellites).

**METHOD**. *hRad30A* has 10 coding exons and an unexpressed exon I 5.8 kb upstream from the first coding exon. To investigate the basis for the alternative splicing we subjected the 5' end of the gene to analysis by a Genome Cryptographer (GC) program[6]. This is an exhaustive genome landscaping mapping that identifies repetitive element density and allows correlation between these elements and functional activity.

**RESULTS AND DISCUSSION**. GC analysis indicated that many of coding regions of *hRad30A* were regions of low complexity. This was anticipated since these should be informative for gene product synthesis and function. However, the long, 5.8kb intron I also contained at least 1kb of unique sequence with no detectable repetitive DNA, unlike the flanking regions, and a high frequency of CpG islands. This indicates that the intronic region has escaped invasion by repetitive DNA and probably contains important coding or regulatory regions. This region may therefore contain control elements for the tissue-specific splicing we previously observed (5), and will be the subject of further sequence and functional analysis.

**ACKNOWLEDGEMENTS**. Work supported by NIEHS grant number ES/CA08061 and the Ellison Foundation, with additional support from the XP Society.

**REFERENCES**

1. Johnson, R.E., Kondratick, C.M., Prakash, S., and Prakash, L. (1999) Science 264, 263–265.
2. Masutani, C., Kusumoto, R., Yamada, A., Dohmae, N., Yokol, M., Yuasa, M., Araki, M., Iwa, S., Takio, K., and Hanoaka, F. (1999) Nature. 399, 700–704.
3. Johnson, R.E., Washington, M.T., Prakash, S., and Prakash, L. (2000) Science 275, 7447–7450.
4. Matsuda, T., Bebenek, K., Masutani, C., Hanoaka, F., and Kunkel.T.A. (2000) Nature. 404, 1011–1013.
5. Thakur, M., Wernick, M., Collins, C., Limoli, C., Crowley, E., and Cleaver, J.E. (2001) Genes, Chrom. Cancer 32, 222–235.
6. Collins, C., Volik, S., Kowbel, D., Ginzinger, D., Ylstra, B., Cloutier, T., Hawkins, T., Predki, P., Martin, C., Wernick, M., Kuo, W.L., Alberts, A., and Gray, J.W. (2001) Genome Res. 11, 1034–1042.
